# Artificial Intelligence in Advancing Inflammatory Bowel Disease Management: Setting New Standards

**DOI:** 10.3390/cancers17142337

**Published:** 2025-07-14

**Authors:** Nunzia Labarile, Alessandro Vitello, Emanuele Sinagra, Olga Maria Nardone, Giulio Calabrese, Federico Bonomo, Marcello Maida, Marietta Iacucci

**Affiliations:** 1 Department of Gastroenterology, National Institute of Gastroenterology—IRCCS “Saverio de Bellis”—Castellana Grotte, 70013 Bari, Italy; 2 Department of Medicine and Surgery, University of Enna ‘Kore’, 94100 Enna, Italy; alessandrovitello86@gmail.com (A.V.); marcello.maida@hotmail.it (M.M.); 3 Gastroenterology Unit, Umberto I Hospital, 94100 Enna, Italy; federicobonomo1@gmail.com; 4 Gastroenterology Unit, Fondazione Istituto San Raffaele Giglio, 90015 Cefalù, Italy; emanuelesinagra83@googlemail.com; 5 Gastroenterology Unit, Department of Public Health, University of Naples Federico II, 80131 Naples, Italy; olga.nardone@libero.it; 6 Gastroenterology Unit, Department of Clinical Medicine and Surgery, University of Naples Federico II, 80131 Naples, Italy; giulio.calabrese@unina.it; 7 APC Microbiome Ireland, College of Medicine and Health, University College Cork, T12 YN60 Cork, Ireland; miacucci@ucc.ie

**Keywords:** artificial intelligence, inflammatory bowel disease, ulcerative colitis, Crohn’s disease

## Abstract

Introduction: Artificial intelligence (AI) is increasingly being applied to improve the diagnosis and management of inflammatory bowel disease (IBD). Aims and Methods: We conducted a narrative review of the literature on AI applications in IBD endoscopy, focusing on diagnosis, disease activity assessment, therapy prediction, and detection of dysplasia. Results: AI systems have demonstrated high accuracy in assessing endoscopic and histological disease activity in ulcerative colitis and Crohn’s disease, with performance comparable to expert clinicians. Machine learning models can predict response to biologics and risk of complications. AI-assisted technologies like confocal laser endomicroscopy enable real-time histological assessment. Computer-aided detection systems improve identification of dysplastic lesions during surveillance. Challenges remain, including need for larger datasets, external validation, and addressing potential biases. Conclusions: AI has significant potential to enhance IBD care by providing rapid, objective assessments of disease activity, predicting outcomes, and assisting in dysplasia surveillance. However, further validation in diverse populations and prospective studies are needed before widespread clinical implementation. With ongoing advances, AI is poised to become a valuable tool to support clinical decision-making and improve patient outcomes in IBD. Addressing methodological, regulatory, and cost barriers will be crucial for the successful integration of AI into routine IBD management.

## Simple Summary

In this narrative review, we explore the current applications and limitations of artificial intelligence in the field of inflammatory bowel disease. The aim is to provide a comprehensive overview of how AI technologies—such as machine learning and deep learning—are being used in various aspects of IBD care, including diagnosis, disease monitoring, treatment response prediction and personalised medicine. At the same time, this review highlights existing challenges, such as data heterogeneity, lack of standardizations, and ethical concerns, which still limit the full integration of AI into clinical practice. We hope this review will be a guide for future research and development.

## 1. Introduction

Inflammatory Bowel Disease (IBD), which includes Crohn’s Disease (CD) and ulcerative colitis (UC), encompasses chronic idiopathic inflammatory disorders affecting the gastrointestinal tract [1] whose prevalence is forecasted to be nearly 1% of the population in early industrialised countries [2,3,4]. Recurrent episodes of inflammation and ulceration of the bowel characterise the natural history of CD and UC. This can lead to several complications, which impact a patient’s quality of life and healthcare costs due to hospitalisation, surgery, and advanced therapies [5,6,7]. An established and timely diagnosis, personalised treatment strategies, and tight monitoring are critical for mitigating disease morbidity. Given the emerging data, achieving precise IBD management has become a turning point. Cross-sectional imaging, endoscopy, and histology are reliable instruments for correctly diagnosing IBD [8,9]. However, interpreting these studies often relies on subjective human judgement, leading to significant delays, interobserver variability, and/or potential diagnostic discrepancies [10]. Furthermore, the rising incidence of IBD globally [11,12] and the availability of digitalised data have intensified the demand for innovative approaches to facilitate diagnosis and empower clinical management [10,13].

In this scenario, artificial intelligence (AI) can integrate multiple and complex data (i.e., genomics and proteomics) to achieve precise disease management. The main AI applications in IBD encompass therapy response prediction, endoscopic disease activity scoring, drug discovery, and the detection of bowel damage through imaging. AI systems are expected to succeed in a process humans cannot easily perform [14]. This ultimately poises to advance clinical trial recruitment for IBD leading to the design and discovery of novel medications [15].

AI is any technique that allows computers to mimic human intelligence by using logic, decision trees, or machine learning.

Machine learning (ML) methods represent an AI branch of the computational methods needed for complex pattern-recognising data analytics. Hence, they are the best-designed tool for data analysis [13]. This system can analyse various electronic data, such as laboratory values, imaging items, administrative and diagnostic codes, demographics, and billing codes [13].

To date, the most common application of AI in IBD care is computer vision systems, including endoscopy [16]. Literature data on this topic harbour promising findings that support AI’s potential to improve patient care and advance our understanding of the heterogeneous nature of IBD [17].

Based on these premises, we performed a narrative review aiming to summarise the recent advances in the application of AI technologies in IBD endoscopy.

## 2. Methods

The narrative review was conducted using the free PubMed database employing MeSH terms related to IBD, such as “inflammatory bowel disease”, “ulcerative colitis”, “Crohn’s disease”, “mucosal healing”, “endoscopic/histological activity/scores”, “biological therapy”, and “immunosuppressive therapy” separated by the Boolean operator.

## 3. Artificial Intelligence Overview

### Explanation of AI Concepts Relevant to Healthcare

AI applications in healthcare consist of well-designed ML methods for data analysis, such as support vector machines (SVMs), random forests (RFs), and artificial neural networks (ANNs). SVMs are classification methods that plot boundaries between two populations based on a value dataset provided [18]. RFs are binary decision trees trained under supervision to predict a specific dichotomous outcome [19]. ANNs repeat biological neural networks (convolution neural network—CNN), giving output classification following an input furnished by the human: this input is represented by unstructured data like images or videos (i.e., endoscopic and cross-sectional imaging), whose characteristics, such as brightness and colour, are recognised by the algorithm [17]. Humans can label the input items according to the classifications or annotations as clinical outcomes or conditions in a “supervised training” process. Data is modelled to a particular output of choice, representing disease grading or phenotype, a clinical outcome, or another event [17]. During this phase, the system accurately builds relationships (neural networks) between data, thus associating specific outcomes. Otherwise, when the input is not labelled, the training is “unsupervised”: in this setting, the AI system groups the inputs without associating them with an outcome [17].

Natural language processing (NLP), which is based on the same process but with a different application, represents an additional ML feature: it enables machines to read and understand text, changing our ability to extract information and predict patient outcomes [17]. AI has the potential to relieve healthcare providers from the laborious and mechanical components of healthcare, and it also has the potential to discover new disease insights to fill many gaps in delivering high-quality care [17].

The complex and individualised information delivered through AI systems can help pursue a personalised approach for each patient, which could receive a more subtle evaluation than it could be from humans [17].

## 4. AI Applications in Inflammatory Bowel Disease

**Figure 1:**
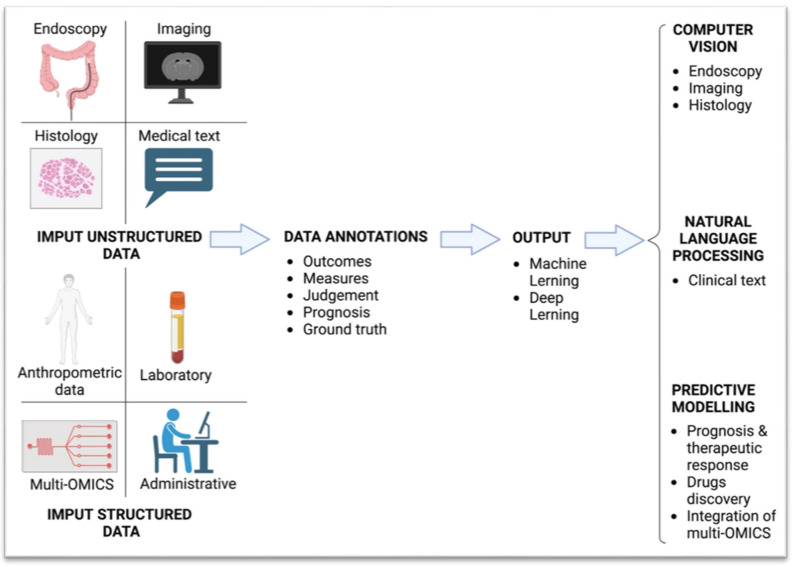
Artificial intelligence model workflow.

AI applications range from determining the risk of developing IBD to helping to assess mucosal activity or discover dysplasia through endoscopy or imaging, supporting histopathological reporting, forecasting the disease course and determining the effectiveness of biological treatments (Figure 1). Table 1 summarises the main studies and research articles that have explored AI applications in IBD management.

### 4.1. Endoscopic Diagnosis and Assessment of UC Enabled by AI

Accuracy and reproducibility are crucial in treating-to-target IBD management [86]. However, diagnosis and assessment of disease activity require highly specialised IBD endoscopists. Despite their expertise, the inter-observer variability of endoscopists remains an issue, given the low reproducibility of most endoscopic tools available [87]. AI’s role becomes fundamental because improving diagnostic accuracy should reduce interobserver variability.

In recent years, several studies have developed and verified the applicability of ML algorithms to classify UC endoscopic disease activity using high-quality colonoscopy frames and videos. One of the first studies was conducted by Sasaki et al. [20] in 2003: 133 colonoscopy images were obtained from 55 UC patients, where the Matts score was characterised using mucosal redness parameters. The degree of mucosal redness was proportional to the histological microvascular bed and, therefore, to disease activity. This algorithm was able to differentiate Matts grades with high sensitivity and specificity.

Across the years, technology has progressively advanced and more sophisticated AI systems have been tested and have demonstrated excellent performance and good agreement with the experts in assessing MES and UCEIS [23,24,25,26].

Stidham et al. have also applied the concept of a weighted AI-based endoscopic score. with the final objective to homogenise endoscopic evaluation in randomised control trials (RCTs) [88]. They trained a CNN to score multiple frames of left colonic UC patients from the UNIFI study of ustekinumab and develop a cumulative disease score (CDS) based on MES. The CDS showed greater sensitivity and statistical power to detect endoscopic changes than the Mayo Endoscopic Score

Despite the high agreement rate between the AI systems and the endoscopic scores, the labelling represents a critical point. AI systems are trained by humans who score images according to systems lacking optimal inter-observer reliability [87].

In an attempt to overcome this issue, in a pilot study on 29 UC patients and six control patients conducted by Bossuyt et al. [22], an operator-independent computer-based algorithm (Red Density^®^, Pentax, Tokyo, Japan) based on the red channel of the red–green–blue pixel values and vessel pattern detection was tested. This score significantly correlated with both endoscopic and histological scores, achieving good agreement with MES, Ulcerative Colitis Endoscopic Index of Severity (UCEIS) and Robarts Histological index (RHI) (r = 0.76, 0.74, and 0.74, *p* < 0.01, respectively).

A further important field of AI is predicting histological activity. In a Japanese multicentre cross-sectional study, Takenaka et al. [27] validated an algorithm to assess endoscopic and histopathological disease activity through UCEIS and Geboes scores. The results of the real-life validation phase were remarkable: high accuracy for both endoscopic and histological remission (90% and 93%, respectively) and good intraclass correlation between CNNs and endoscopists (0.917) and CNNs and pathologists (0.859).

The same group subsequently refined the previous algorithm to assess disease activity directly on videos in a large prospective study involving 770 patients and 900 biopsy specimens. The study showed a sensitivity of 97.9% and a specificity of 94.6% for predicting histologic remission [89].

Recently, using 795 videos from a study on Mirikizumab, ref. [31] a CNN was developed to assess mucosal activity using MES and UCEIS [30]. Agreement with expert readers was excellent for both MES and UCEIS (0.844 and 0.855, respectively). In particular, model performance was best for MES scores 0 and 3 and worst for MES scores 1 and 2.

Iacucci et al. [31] developed a CNN to evaluate endoscopic and histological activity and clinical outcomes on 1090 videos in WLE and VCE of the multicentre Picasso study. The results for WLE were a sensitivity of 72%, a specificity of 87% and AUROC of 0.85 and 79%, 95%, and 0.94 for virtual chromoendoscopy (VCE).

All these studies clearly show how AI will improve clinical practice thanks to its ability to simultaneously analyse multiple datasets and help the doctor in the diagnosis and correct characterisation of disease activity.

This has also been confirmed by systematic reviews and meta-analyses which have highlighted how AI has high accuracy in the diagnosis of endoscopic remission for both MES and UCEIS, both for single frame and video [90,91,92].

### 4.2. Endoscopic Diagnosis and Assessment of Disease Activity in CD Enabled by AI

The CD is characterised by various endoscopic patterns that can belong to different locations, including the ileum. Hence, AI is not ideal for reproducing SES-CD and CDEIS.

However, AI-assisted video capsule endoscopy (VCE) can aid in detecting small bowel ulcers and, importantly, help overcome limitations of VCE itself, which is a time-consuming examination and subject to wide inter-observer variability [38,41,93,94].

A systematic review with meta-analysis, which included both IBD and non-IBD patients, concluded that the overall accuracy of AI-enhanced VCE in detecting ulcers or bleeding is 95.4% [95].

Charisis and Hadjileontiadis [32] created a novel filtering process (hybrid adaptive filtering—HAF) to extract lesion characteristics of VCE images and a Differential Lacunarity (DLac) analysis was applied on the HAF-filtered images. The HFA DLac system showed higher accuracy for severe lesions (93.8%) and slightly lower for mild lesions (81.2%).

A CNN created by Aoki et al. [35] to detect CD ulcers or erosions using 5360 VCE images completed the evaluation in just under 4 min. It had a sensitivity of 88%, a specificity of 99%, and an overall AUROC of 0.99.

Further studies have validated new computer-assisted methods to detect ulcers and erosions in the small intestine with accuracy rates around 95% [33,34].

Klang et al. found that ANNs trained on VCE images can detect small bowel ulcers with approximately 95% accuracy and identify the presence of non-obstructive strictures [39].

In 2021, Barash et al. confirmed that CNN-assisted VCE readings have a high potential for classifying CD ulcers [40]. Subsequently Kratter et al. developed a combined model for two different capsules (PillCam Crohn and PillCam SB3, Medtronic, Osaka, Japan), with good performance in classifying grade 1 and grade 3 ulcers, with AUC of 0.99 [37].

Ferreira et al. developed a CNN for the identification of panenteric ulcers in the PillCam^TM^ (Medtronic, Dublin, Ireland) Crohn’s capsule, which presented high sensitivity (98%), specificity (99%) and accuracy (99%), as well as a great ability to differentiate ulcers and erosions [36].

Ding et al. [41] also confirmed all these data, reporting that AI significantly reduced gastroenterologists’ reading time from 96.6 min to 5.9 min, with no difference in performance. In another study, AI reduced reading time from 12.2 min to 3.1 for experienced examiners and from 20.7 to 5.2 for trainees without affecting overall accuracy [42].

Brodersen et al., in a recent prospective multicentre study on patients with CD, confirmed the reduction in reading time thanks to AI, without reducing diagnostic accuracy [38].

Overall, these studies demonstrate that integrating CNN models significantly accelerates and improves the accuracy of VCE, thereby greatly facilitating the diagnosis and monitoring of Crohn’s disease patients.

### 4.3. AI Drives Advanced Endoscopic Technologies

In recent years, novel endoscopic techniques have enabled real-time histological assessment of the colonic mucosa during endoscopic procedures. This approach requires specialised endoscopists and advanced interpretative skills. Furthermore, operator dependence on these tools should not be underestimated. Confocal laser endomicroscopy (CLE) represents one of these systems capable of assessing deep mucosal healing (up to 1250 time-fold magnification). Quénéhervé et al. [43] studied the potential of artificial intelligence-guided CLE diagnosis in a retrospective analysis of IBD patients (23 CD and 27 UC) and healthy subjects (*n* = 9). Excellent accuracy was obtained for diagnosing IBD (sensitivity and specificity of 100%) and differentiating UC from CD (sensitivity of 92%, specificity of 91%).

Another advanced endoscopic imaging technique is endocytoscopy (up to 1390 times magnification). Maeda et al. [44] created a CAD system to automatically determine the histologic activity score by endocytoscopy (defined by a Geboes score ≥of 3.1) using 12,900 endocytoscopic images from 87 UC patients. The results showed a sensitivity, specificity, and accuracy of 74%, 97%, and 91%, respectively, using the pathologist’s interpretation as the gold standard.

Moreover, a new endoscope with single short-wavelength monochromatic LED illumination (Fujifilm, Tokyo, Japan) has recently been developed, and it can visualise pericryptal capillaries to a depth of approximately 50–200 μm in real time and without the need for contrast agents. Bossuyt et al. [45] described a new CAD technique to assess histologic remission on images obtained from this new endoscope in a prospective study of 58 UC patients. The CAD algorithm successfully predicted the histologic remission of UC with high accuracy (86%) compared with standard endoscopic scoring systems (MES 74%, UCEIS 79%).

Recently, Sinonquel et al. evaluated the accuracy of a CAD system for estimating histological activity in UC, based both on WLE and on single-wavelength endoscopy (SWE). SWE-CAD exceeded the accuracy of WLE-CAD; it showed an accuracy of 95.2%, sensitivity of 96.4%, and specificity of 92.9% [46].

Studies cited in the previous three subchapters highlight how AI achieves high accuracy in identifying endoscopic disease activity, mucosal healing, and even subtle features that might otherwise escape the human eye. These systems can offer a standardised and objective score of disease severity, reducing interobserver variability, a major problem in current clinical practice. By automatically assigning endoscopic scores, AI streamlines both clinical care and research protocols.

Furthermore, real-time AI-assisted endoscopy can contribute to immediate decision-making during colonoscopy, highlighting areas of inflamed mucosa, suggesting biopsy sites, or even predicting histological remission.

It is likely that future clinical guidelines will recommend the use of validated AI tools as a complement to human interpretation, especially in centres with access to high-resolution imaging and digital infrastructure. To ensure safety and efficacy, guidelines could also define criteria for validating AI tools, including required accuracy parameters, training data diversity, and integration with existing electronic health record systems. The integration of AI into the endoscopic assessment of IBD will never replace the physician, but rather will improve accuracy, consistency, and efficiency, ushering in a new era of data-driven, personalised care.

### 4.4. Personalising Therapy Through AI: Tailoring Treatment for Optimal Patient Outcome

#### 4.4.1. AI in Predicting Response to Therapy

Patients with IBD have different responses to therapies, and predicting the therapeutic response to a specific drug could help obtain deep healing in a shorter time and reduce healthcare-associated costs.

Over the years, predictive models in IBD have been developed using statistical regression, which cannot analyse more complex data structures such as repeated measurements. It has been shown that AI can overcome these limitations. In the thirteen studies evaluated in the systematic review conducted by Nguyen et al., ML-based methods were found to be more effective than traditional statistical methods in predicting treatment response, disease activity, and long-term complications [96].

Deep learning models were developed to predict response to anti-TNF therapy: Con et al. used the CRP biomarker to predict CD remission after treatment demonstrating that ML methods had superior predictive capabilities than conventional statistical model [AUROC; 0.754 (95% CI: 0.674–0.834) vs. 0.659 (95% CI: 327 0.562–0.756); *p* = 0.036] [47].

Popa et al. [48], instead, used an AI system processing data relating to the clinic and endoscopy of UC patients in anti-TNF therapy. This system showed a well-performing receiver operating character (ROC) curve (PPV 100%, NPV 100%; *p* < 0.001), with a good ability to accurately differentiate those who will reach clinical remission from those who do not.

An ML model developed by Park et 337 al. found that non-durable responses to anti-TNFs in CD patients was related to elevated 338 expression of DPY19L3 and GSTT1 genes [49].

Similar studies have been performed to predict response to ustekinumab in CD using the UNITI-1 and two studies [50]. Predictions of remission at week 42 had a sensitivity and specificity of 0.79 and 0.67 using week eight post-treatment data, although baseline pre-treatment data had poor predictive value (0.63 and 0.64, respectively). Response to ustekinumab in CD patients was also predicted by another ML model using four genes: HSD3B1, MUC4, CF1, and CCL11 [51].

Among the studies that used an ML model to predict response to thiopurines is that of Waljee et al., in which the AUROC for remission predicted by the algorithm was 0.79 [52].

Regarding vedolizumab, Waljee et al. applied ML models on GEMINI 1 and 2 data for UC patients to predict clinical remission at week 52 with a sensitivity and specificity of 0.76 and 0.71, respectively [53]. Furthermore, the model could predict therapeutic failure in 95.3% of patients using week 6 data and in 88% of cases using only pre-treatment data. Meanwhile, Dulai et al. applied another model on CD patients treated with vedolizumab. In the first phase [54], data from the GEMINI 2 study were used to evaluate whether differences in remission rates were associated with different drug concentrations; in the second validation phase [55], data from the GETAID and VICTORY studies were used to identify patients who could benefit from an increase in the drug dose; finally, the risk of surgery or hospitalisation during vedolizumab therapy was assessed.

A platform developed by Venkatapurapu et al. to predict endoscopic remission and mucosal healing after vedolizumab showed good sensitivity and specificity (80–75% and 69–70%, respectively) [56].

Among the studies that have evaluated therapeutic response to biologics using AI, the Endo-Omics study, analysed in vivo pCLE images and ex vivo molecular labelling of mucosal biopsies [57]. The following factors demonstrated predictive ability for response to therapy: vessel tortuosity, crypt morphology, and fluorescein leakage in vivo and increased binding of a labelled biologic ex vivo.

The therapeutic landscape for IBD, and particularly for moderate-to-severe CD, has evolved rapidly over the past decade. As highlighted in the most recent ECCO guidelines [97], physicians now have access to a growing arsenal of therapeutic options, including anti-TNF agents, anti-integrins, IL-12/23 inhibitors, JAK inhibitors, and S1P modulators. These new therapeutic options offer the possibility of more personalised and effective treatment, but at the same time complicate the therapeutic decision-making process. Each of these therapies has unique efficacy profiles, safety considerations, mechanisms of action, and potential responses specific to each patient. Deciding which agent to use first, how to sequence treatments is becoming increasingly challenging, especially given the heterogeneity of CD.

In this context, ML trained on large datasets that include clinical characteristics, genetic markers, past medical history, and clinical outcomes could help physicians identify the most appropriate therapy for individual patients. AI can integrate multidimensional data to generate predictive models that estimate the likelihood of treatment response or adverse events, offering a level of precision that goes beyond traditional clinical judgement.

#### 4.4.2. AI in Predicting the Course of the Disease by Determining the Histological Activity

Histological remission has gradually become a therapeutic target, and AI enables us to make histological assessments in real time without requiring biopsies. As previously reported, Takenaka et al. used 40,758 colonoscopy images and 6885 histological images to predict histological remission (HR, Geboes score < 3) with 93% accuracy [27].

This is important because a prospective follow-up study demonstrated that both endoscopic and histological remission were associated with a significant reduction in hospitalisation, colectomy, steroid use, and clinical relapse (*p* < 0.001) [28].

Iacucci et al. [61] applied the simplified neutrophil-only Paddington International Chromoendoscopy Score virtual histological remission index (PHRI) to a computer-assisted diagnostic system. When comparing the AI-generated assessment results with those generated by pathologists, the AI model was highly sensitive and specific in determining the presence of neutrophils. Gui et al. [62] also evaluated the applicability of PHRI in an AI system on 614 biopsies of 307 UC patients, achieving excellent results in the system’s ability to signal the presence or absence of neutrophils (sensitivity 78%, specificity 91.7%, accuracy 86%). Furthermore, mucin depletion is one of the histological indicators of clinical relapse in UC. Ohara et al. [60] created a DL to quantify goblet cell mucus areas. Their model has achieved an accuracy of 0.97, allowing us to predict clinical relapse in UC.

Maeda et al., in their study cited above, in which they applied AI to endocytoscopy [45], also found that clinical recurrence at 12 months was higher in the group with histologically active disease (28.4 vs. 4.9%, *p* < 0.001) [44].

Two studies [58,59] developed DL algorithms to quantify eosinophils in colonic biopsies, since eosinophils have been associated with disease course and therapeutic response. These models have achieved accuracies between 0.85 and 0.89.

These studies show that AI can become a valuable tool in clinical practice to identify those patients who are most at risk of recurrence through AI-guided assessment of histological activity. However, more standardised and large-scale studies are needed to validate these results.

#### 4.4.3. AI for Continuous Monitoring of Disease Activity and Patient Self-Assessment

Monitoring symptoms and biomarkers is required in IBD patients to evaluate the progress of inflammation and the therapeutic response. Therefore, the need for digital health technologies (e.g., smartphones, tablets, internet-based devices, and wearable devices) to monitor therapy is increasing [98]. A review about health apps useful in IBD identified eleven apps powerful in the therapeutic management of hospitalised patients [99].

Additionally, a natural language processing (NLP)-based chatbot was created to categorise IBD patients’ electronic messages into various categories (e.g., questions about medications or lab tests). The agreement between the algorithm and clinicians was 95% [63].

Jagannath et al. [100] created a sensor capable of monitoring biomarkers present in the sweat of IBD patients.

Furthermore, AI systems that interpret endoscopic images facilitate remote telemedicine consultations and simplify and speed up trainee training [101].

#### 4.4.4. AI for Evaluation of Histological Activity and Diagnosis

AI has made significant strides in improving the histological assessment of IBD patients. A recent meta-analysis [102], including twelve studies, showed how AI evaluates the histological activity objectively and accurately, overcoming the inter- and intraobserver variability.

Rymarczyk et al. developed a DL model to predict the Global Histology Activity Score [GHAS] for CD and a Geboes histopathology score for UC with accuracies ranging from 65% to 89% [71].

Matalka’s automated system [72] achieved 98% precision in diagnosing IBD by assessing crypt architecture and mucosal damage. A landmark deep learning algorithm identified eosinophils in ulcerative colitis, showing strong agreement with pathologists (ICC 0.81–0.92), though recently, attention has been focused on neutrophils.

The absence of neutrophil infiltration is crucial for assessing remission, Ohara et al. developed an AI system capable of accurately quantifying and localising neutrophils in UC biopsy specimens to facilitate histological assessment. Their model achieved a performance of 0.77, 0.81, and 0.79 for precision, recall, and F-score, respectively [73].

The PICaSSO Histologic Remission Index has indeed simplified histological scoring by focusing on neutrophils, displaying robust correlations with endoscopic scores (ICC 0.84) [62]. A deep learning strategy by Gui et al., analysing 614 biopsies, accurately predicted histological remission with 80% validation accuracy [62]. Further refinement by Del Amor [74] introduced multi-instance learning frameworks capable of distinguishing ulcerative colitis activity with high sensitivity and specificity.

In 2022, Peyrin-Biroulet et al. [75] developed an AI-based scoring system aligned with the Nancy Histopathology Index, achieving ICC 0.87. Najdawi’s 2023 CNN model advanced segmentation and quantification of histological features, with a random forest classifier predicting remission at 97% accuracy [76].

For CD, Kiyokawa’s [77] DL model predicted postoperative recurrence with an AUROC of 0.995, identifying adipocyte shrinkage and mast cell infiltration as key features.

Rubin et al. have recently developed a novel DL-based AI to simplify the histopathological evaluation using the Nancy index in UC patients. Their AI model reached high correlation with histopathologists’ assessments [78].

While these advancements show great potential in improving diagnostic precision and supporting clinical decision-making, further refinement and validation are necessary before routine adoption in clinical and research settings.

#### 4.4.5. The Role of AI in Detecting Colitis-Associated Neoplasia

IBD patients have a significantly increased risk of colorectal cancer compared with the general population. In 2020, Maeda et al. [68] reported the first case report of AI-assisted detection of colitis-associated neoplasms in a 72-year-old patient with an 18-year history of colitis.

Furthermore, EndoBRAIN-EYE (Cybernet Systems, Tokyo, Japan) is an artificial intelligence-based system previously used in non-IBD patients [69].

Yamamoto et al. [70], in a pilot model, tested an AI system for characterising neoplasia occurring in IBD. The model classified lesions into two groups: “adenocarcinoma/high-grade dysplasia” and “low-grade dysplasia/sporadic adenoma/normal mucosa.” When compared against experts and non-experts, the AI diagnostic accuracy of the AI model was higher (nonexperts, 77.8%; experts, 75.8%; AI model, 79.0%).

In 2023, Vinsard et al. [79] conducted a prospective study on 3437 surveillance colonoscopies to retrain for IBD dysplasia detection and AI-system-based sporadic adenomas. The system increased sensitivity from 50% and accuracy from 64% to 96.8%.

More recently, a specific CADe model for IBD dysplastic lesions detection has been developed by Abdelrahim et al. [80], showing a lesion detection rate of 90.4% and a negative predictive value of 94.3% in small sample real-time validation.

#### 4.4.6. AI in Predicting Disease Progression, Complications, and Risk Stratification

AI can analyse complex multi-omics data and bring together genetic and microbial data to help clinicians make clinical decisions. ML models using RNA expression levels from whole blood samples performed risk stratification by identifying high- and low-risk groups for future dose escalations in both CD (75% vs. 35%) and UC (60% vs. 20%) [64].

ML models were also created to effectively predict 1-year relapse risk in CD after surgery [65], and two ML models have also been developed to predict the risk of surgery in patients with CD. Using laboratory variables, Stidham et al. [81] found that anti-TNF therapy, unlike corticosteroid use and hypoalbuminemia, was the strongest predictor associated with a lower risk of surgery within one year. Dong et al. [66] instead used different clinical variables and their ML model showed higher accuracy and precision than the statistical model.

A neural network developed for severe acute ulcerative colitis (ASUC) combined data from a pool of 3391 miRNA candidates and clinical factors. It effectively distinguished medical responders from non-responders with 97% accuracy; the miRNA-only model had 94% accuracy [67].

Further research demonstrated that an AI model (EndoBRAIN-UC) could stratify the risk of clinical relapse in UC patients in clinical remission. This model classified patients into two cohorts detected by AI: active disease and healing disease. The first group experienced clinical relapse in 28.4%, compared to 4.9% in the second group (*p* = 0.01) [83]. Omori et al. confirmed the efficacy of EndoBRAIN-UC by showing a sensitivity of 74.2% and a specificity of 93.8% for the histological diagnosis of remission [84].

Similarly, Kuroki et al. [85] developed an AI-based system to diagnose “vascular healing” by distinguishing two groups “vascular-active or vascular-healing” and establishing the role of AI-based vascular healing in predicting the outcomes of UC patients. Also in this case, clinical recurrence was higher in the vascular-active group.

The role of endomicroscopy in predicting disease outcomes has been explored by Iacucci et al. [57]. In the Endo-Omics Study, they explored the use of endomicroscopy morphology and fluorescein leakage together with the ex vivo evaluation of tissue biologics binding to predict the response to a specific treatment. In UC, the samples with higher drug binding were associated with a better response to treatment. Ultimately, AI-based risk stratification studies have been conducted regarding the predictive role in barrier healing for major adverse outcomes in patients with UC [82]. The evaluation Claudin-2, Occludin, and JAM-A has been found to be effective in predicting clinical outcomes over 12 months. Multicentre prospective studies are currently exploring this field further.

## 5. Challenges, Limitations, and Implementation

### 5.1. Methodological Issues of AI Application to IBD

**Figure 2:**
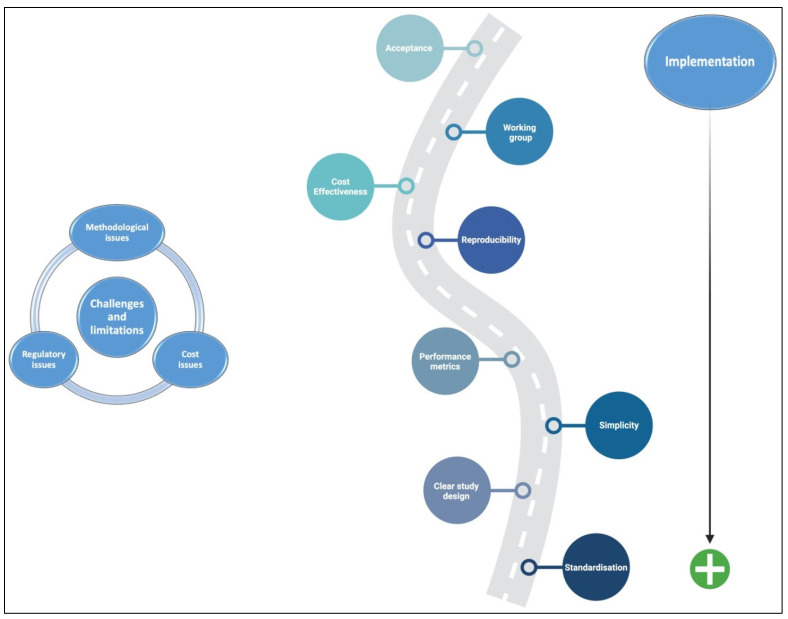
Suggested roadmap for the challenges, limitations, and implementation of AI in clinical practice and clinical trials.

Even though AI has a lot of potential to help with IBD diagnosis and management, research may run into several challenges that limit the complete integration of this tool into routine clinical practice [103]. Various methodological challenges represent an important issue in AI, undoubtedly related to using small datasets, a lack of external validation, and problems with inconsistent or unclear study designs, reporting, and delivery [104]. Additionally, because tagged data forms the basis of most existing AI models, interpretability depends on the accuracy of the observer who labelled the “gold standard” data [105,106]. Figure 2 summarises a suggested roadmap for the challenges, limitations, and implementation of AI in clinical practice and clinical trials.

A significant percentage of AI models used in healthcare contexts have undergone internal validation; nonetheless, to prevent overfitting, these models have to be externally validated on various cohorts [105]. Furthermore, researchers are unwilling to report bad AI algorithms, and journals are unlikely to publish these negative findings. Hence, there is a large amount of publication bias in the existing literature [10,107].

Every AI system needs high-quality data during training and validation to provide accurate predictions. However, healthcare data frequently lack standardisation, are inconsistent, incomplete, and of low quality (mainly retrospective), often making these clinical applications untrustworthy in the eyes of clinicians [103]. Standardised methods for acquiring endoscopic pictures and videos are lacking, particularly during automated endoscopic scoring. In addition, electronic health record data are sometimes isolated and challenging to access. This makes it harder to create algorithms with broad applications and restricts the quantity of data accessible to train algorithms.

Algorithmic bias remains a major challenge. Indeed, clinical trials often fail to adequately represent or accurately reflect specific groups of patients, such as racial or ethnic minorities or those with more complex IBD presentations (e.g., fistulizing CD, pouchitis, older ages, comorbidities, and polypharmacy).

This discrepancy arises when AI models are trained on historically unbalanced datasets, inadvertently reinforcing existing biases and leading to skewed outcomes in patient care and treatment efficacy [108,109,110].

Recently, guidelines for standardising reporting in clinical trials assessing the performance of AI have been developed, such as the CONSORT (Consolidated Standards of Reporting Trials)—AI extension and the SPIRIT (Standard Protocol Items: Recommendations for Interventional Trials)—AI, the latter representing its companion statement for clinical trial protocols [111].

In the future, the use of standardised endoscopic data collected from RCTs will strengthen the development of improved AI-based endoscopic scoring systems [10], even if new guidelines for data collection, storage, and sharing, as well as creating technology capable of combining data from many sources, are necessary to address these problems [112].

Furthermore, to effectively address bias, it is essential to ensure diversity in training datasets, adopt frameworks capable of detecting bias, and apply both adversarial debiasing strategies and fairness-aware machine learning algorithms [113,114].

### 5.2. Regulatory Issues of AI Application to IBD

Currently, privacy and ethical issues regarding AI applications to patients’ data, encompassing genetic, biomarker, and multi-omics information should be considered. To preserve patient anonymity, the healthcare environment is governed by stringent privacy and security laws that differ between organisations, nations, and continents. Due to these limitations, sharing data between institutions for AI analysis can be difficult, and data violations can also have negative effects and legal consequences.

Robust data security measures, such as anonymisation, encryption, strict access controls, and the use of a decentralised federated dataset (aggregating data across multiple servers), provide a practical solution to these challenges while ensuring the protection of patient confidentiality [111].

In addition, bias, transparency, accountability, and interpretability are frequent ethical issues related to AI use in different clinical contexts. Many AI models operate as ‘black boxes’, generating predictions without clear or easily understandable reasoning behind them. As a result, physicians may hesitate to fully trust and accept AI predictions if they are concerned about data bias or unable to comprehend how the algorithm reached its conclusions.

On the other hand, AI algorithm recommendations may not align with patient preferences or acceptance.

Recently, the Food and Drug Administration (FDA) issued preliminary regulatory guidance for the use of AI in clinical research [115], although clear, updated, and standardised guidelines must still be established to ensure that AI can be applied safely and effectively to enhance patient care. This should be performed after proper information is provided to patients and their rights and preferences are respected [116].

Regarding legal concerns, medical devices incorporating AI algorithms should have regulatory approval. However, these approval procedures can be drawn out, complicated, and expensive, hindering their adoption in several clinical settings. Medical physicists (MPs) should be involved as participants in acquiring AI tools, acceptance testing, commissioning, and quality assurance to verify the stated performance concerning the medical device’s intended use, as well as in marketing it through scientific journals and scientific congresses [117].

Furthermore, the rapid evolution of AI poses other pressing regulatory challenges. To ensure that AI tools are safe and effective while remaining relevant, there is growing consensus on the need for more agile validation processes and adaptive study designs. AI systems can continually evolve. This dynamic nature requires validation approaches that go beyond traditional approvals. Regulators and clinical researchers must adopt continuous performance monitoring, real-world testing, and revalidation mechanisms as algorithms evolve over time.

Adaptive study designs, which allow for changes to the course of a study based on interim results, offer a promising path forward. These designs can enable the faster identification of effective AI interventions, timely termination of underperforming tools, and better resource allocation. This flexibility is critical for evaluating AI tools, which must demonstrate not only initial accuracy but also sustained utility in a variety of real-world settings. Furthermore, collaboration between regulatory agencies, academic institutions, and technology developers is essential to create standardised yet flexible evaluation frameworks.

In conclusion, to fully harness the potential of AI in healthcare, validation processes must evolve. Agile, adaptive, and data-driven approaches will be crucial to ensuring that innovative AI tools can be safely and efficiently translated into clinical practice, in step with the technology for which they are designed.

### 5.3. Cost Issues of AI Application to IBD

Cost is another crucial factor to take into account when integrating AI into clinical practice as well as in RCTs. A significant investment in infrastructure, data integration platforms, and a skilled working group composed of data scientists and bioinformaticians is essential for the development and maintenance of AI systems to ensure long-term effectiveness [118].

The cost is further exacerbated by the ongoing need for algorithm validation, continuous training, and compliance with legal regulations [118].

## 6. Future Directions and Conclusions

Although AI has made significant progress, several challenges remain to be addressed in future research. In the coming years, AI assistants are expected to take over much of the data collection process. This will free up valuable time for more in-depth analysis, patient education, and critical thinking. However, AI is probably years away from providing direct healthcare. AI systems are unlikely to gather the vast array of emotional and psychosocial data that clinicians naturally understand and utilise in their decision-making, even in the information extraction domain. Noteworthy is that AI is not expected to replace the personal connection and rapport that form the foundation of the doctor–patient relationship.

Nowadays, research strongly advocates using AI to enhance the quality of IBD diagnosis and management. AI-driven tools can deliver consistent, objective, precise, and faster clinical evaluations, predict treatment outcomes, and elevate the quality of endoscopy across all stages. As AI technology becomes more accessible and integrates larger datasets into its algorithms, these models will inevitably improve.

Continuous research and model advancement should prioritise explainable AI methods, enabling clinicians to comprehend and assess how models reach their conclusions (view inside the “black boxes”), thus strengthening clinical justification and preserving clinician accountability.

Hopefully, AI will increase the quality of RCTs, enabling more precise patient selection, standardised diagnostic test interpretation (thus avoiding central reading), and interpretation of clinical outcomes.

Lastly, addressing the abovementioned challenges requires strong collaboration among healthcare professionals, medical physicists, researchers, regulators, and industries.

## Figures and Tables

**Table 1:** Summary of the main studies where AI has been applied in IBD.

Study	Field of Application	AIM	Outcome
Sasaki et al., 2003 [20]	Endoscopic activity	Matts score was characterised using mucosal redness parameters, considered proportional to the histological microvascular bed and to disease activity	The algorithm was able to differentiate Matts 1 from Matts 2, Matts 2 from Matts 3, and Matts 3 from Matts 4 with high sensitivity and specificity
	WLE		
Kraszewski et al., 2021 [21]	Diagnosis	ML model based on routinely performed laboratory blood, urine, and faecal tests to diagnose IBD	The model could diagnose CD and UC with an average accuracy of 97% and 91%, respectively
Bossuyt et al., 2020 [22]	Endoscopic activity	Application of a new algorithm (Red Density) based on the red channel and vessel pattern detection on UC patients	The algorithm significantly correlated with MES, UCEIS and RHI (r 0.76, 0.74, and 0.74, *p* < 0.01, respectively)
	WLE		
Stidham et al., 2019 [23]	Endoscopic activity	Analysis of DL for distinguishing moderate to severe UC from remission compared with multiple expert reviewers	The CNN demonstrated excellent performance in distinguishing MES 0–1 from MES 2–3 and good agreement between expert reviewers (κ = 0.86)
	WLE		
Fan et al., 2023 [24]	Endoscopic activity	Application of DL for objective scoring of endoscopic images and videos in UC patients	The CNN exhibited good accuracy for MES and UCEIS, with a very good agreement (k 0.8) with endoscopists’ scores
	WLE		
Ozawa et al., 2019 [25]	Endoscopic activity	Application of a CNN to evaluate MES in endoscopic pictures from UC patients	The CNN demonstrated a high level of performance with an AUROC of 0.86 and 0.98 for identifying Mayo 0 and 0–1
	WLE		
Takabayashi et al., 2024 [26]	Endoscopic activity	Application of an AI system to assess endoscopic severity of UC	The correlation coefficients between IBD expert endoscopists and the AI of the evaluation results were all higher than 0.95
	WLE		
Takenaka et al., 2020 [27]	Endoscopic/histological activity	Application of a DNN to assess both endoscopic (UCEIS) and histopathological (Geboes score) disease activity	The DNN showed 90% and 93% accuracy for endoscopic and histological remission, respectively; the intraclass correlation coefficients between DNN and experienced endoscopists were 0.917 and 0.859, respectively
	WLE		
Takenaka et al., 2022 [28]	Endoscopic/histological activity	The same group refined the previous algorithm to assess disease activity directly on videos	For predicting histological remission, the DNUC had a sensitivity of 97.9% and a specificity of 94.6%. The intraclass correlation coefficient between DNUC and experts for endoscopic scoring was 0.927
	WLE		
Yao et al., 2021 [29]	Endoscopic activity	To pilot a fully automated video analysis system for grading UC endoscopic disease	The CNN performed better in automatically scoring the local high-resolution video (κ = 0.84) but less well in the unadjusted analysis of the external patient cohort
	WLE		
Gottlieb et al., 2021 [30]	Endoscopic activity	Application of a CNN system to assess mucosal activity according to MES and UCEIS on videos	Agreement with expert readers was excellent for both MES and UCEIS (0.844 and 0.855, respectively). Model performance was best for MES scores 0 and 3 and worst for MES scores 1 and 2
	WLE		
Iacucci et al., 2023 [31]	Endoscopic/histological activity	Application of a new CNN to evaluate endoscopic and histological activity on videos in WLE and VCE of the multicentre Picasso study	The algorithm showed a sensitivity, specificity and AUROC of 72%, 87% and 0.85 for WLE, and of 79%, 95% and 0.94 for VCE
	WLE-advanced imaging		
Charisis and Hadjileontiadis, 2016 [32]	CE	Application of an AI system (HFA DLac) for describing and detecting CD-associated lesions in CE	The accuracy ranged from 81.2% in mild lesions to 93.8% in severe lesions (total 90.5%)
Fan et al., 2018 [33]	CE	Application of a CNN to detect small intestinal ulcer and erosion in CE	Ulcer and erosion detection reached a high accuracy of 95.16% and 95.34%, sensitivity of 96.80% and 93.67%, and specificity of 94.79% and 95.98%, correspondingly, an AUROC of 0.98 in both of the network
Afonso et al., 2022 [34]	CE	A CNN for the automatic identification of small intestinal ulcers and erosions	The model was able to detect and distinguish ulcers and erosions with an accuracy of 95.6%, sensitivity of 90.8%, and a specificity of 97.1%
Aoki et al., 2019 [35]	CE	Application of a CNN created to detect CD ulcers or erosions on CE images	The evaluation was completed in just under 4 min with a sensitivity of 88%, a specificity of 99%, and an overall AUROC of 0.99
Ferreira et al., 2022 [36]	CE	An AI algorithm for the automatic detection of ulcers and erosions of the small intestine and colon in PillCam™ Crohn’s Capsule images	The model had a sensitivity of 98.0% and a specificity of 99.0%. The overall accuracy of the network was 98.8%. The AUROC for detection of ulcers and erosions in PCC images was 1.00
Kratter et al., 2022 [37]	CE	Application of a combined model for two different capsules	The combined model achieved an average AUC of 0.99 and average mean patient accuracy of 0.974
Brodersen, 2024 [38]	CE	Application of the deep learning solution AXARO on panenteric capsules endoscopy	AXARO reduced the initial review time maintaining high diagnostic accuracy
Klang et al., 2020 [37]	CE	Evaluation of a DL algorithm for the automated detection of small-bowel ulcers in CD on CE	ANNs trained on CE images can detect small bowel ulcers with approximately 95% accuracy
Klang et al., 2021 [39]	CE	DL applied on CE images for identification of CD intestinal strictures	DL provided excellent differentiation between strictures vs. normal mucosa, and strictures vs. ulcers
Barash et al., 2021 [40]	CE	Development of DL algorithm for automated grading of CD ulcers on CE	CNN-assisted CE readings have high potential in classifying ulcers in CD
Ding et al., 2019 [41]	CE	Development of a CNN-based algorithm to assist in the evaluation of CE images	The CNN identified abnormalities with 99.90% sensitivity. The mean reading time per patient was 96.6 ± 22.53 min by conventional reading vs. 5.9 ± 2.23 min by CNN
Aoki et al., 2020 [42]	CE	To examine if AI systems can reduce the reading time of endoscopists without decreasing the detection rate of mucosal breaks	AI reduced reading time from 12.2 min to 3.1 for experienced examiners and from 20.7 to 5.2 for trainees, without affecting overall accuracy
Quénéhervé et al., 2019 [43]	CE	Evaluation of the potential of AI-guided CE diagnosis in a retrospective analysis of IBD patient	Excellent accuracy was obtained for the diagnosis of IBD (sensitivity and specificity of 100%) and for the differentiation of UC from CD (sensitivity of 92%, specificity of 91%)
Maeda et al., 2019 [44]	Histological activity	Evaluation of a CAD system to predict persistent histologic inflammation from endocytoscopy, validated on UC patients	The CAD system showed a sensitivity, specificity, and accuracy of 74%, 97%, and 91%, respectively; it also predicted clinical recurrence at 12 months, finding that this was at a higher rate in the AI-histologically active group (28.4 vs. 4.9%, *p* < 0.001)
	Advanced imaging		
Bossuyt et al., 2021 [45]	Histological activity	Application of a CAD technique to assess histologic remission on images obtained from a Single-wavelength endoscope	The CAD algorithm successfully predicted histologic remission of UC with high accuracy (86%)
	Advanced imaging		
Sinonquel et al., 2024 [46]	Histological activity	Evaluation of histological activity using a CAD system based on either WLE or SWE.	SWE-CAD exceeded the accuracy of WLE-CAD; it showed an accuracy of 95.2%, sensitivity of 96.4%, and specificity of 92.9%
	WLE-advanced imaging		
Con et al., 2021 [47]	Response to therapy	A deep learning model developed to predict response to anti-TNF therapy in CD patients, using the CRP biomarker	ML methods showed stronger predictive performance than the conventional statistical model (AuROC; 0.754 [95% CI: 0.674–0.834] vs. 0.659 [95% CI: 0.562–0.756]; *p* = 0.036)
Popa et al., 2020 [48]	Response to therapy	AI algorithm to predict clinical remission in UC patients on anti-TNF therapy, using clinical and endoscopic data	This system showed a well-performing ROC curve (PPV 100%, NPV 100%; *p* < 0.001), with ability to differentiate those who will achieve clinical remission from those who will have active disease
	WLE		
Park et al., 2022 [49]	Response to therapy	A ML model using transcriptome imputed from genotypes to predict non-durable response to anti-TNF treatment in CD	Imputed gene expression characteristics in machine learning models successfully predicted a non-durable response to anti-TNF
Waljee et al., 2019 [50]	Response to therapy	A ML models in prediction response to ustekinumab in CD patients	Predictions of remission at week 42 had a sensitivity and specificity of 0.79 and 0.67 using week 8 post-treatment data
He et al., 2021 [51]	Response to therapy	A ML model based on the expression of four gene to predict response to ustekinumab in CD patients	The AuROC of the model for the training and testing datasets was 0.746 and 0.734 respectively
Waljee et al., 2017 [52]	Response to therapy	ML model to predict response to thiopurines	The AuROC for remission predicted by the algorithm was 0.79
Waljee et al., 2018 [53]	Response to therapy	ML models for UC patients to predict clinical remission to vedolizumab at week 52	The model showed a sensitivity and specificity of 0.76 and 0.71, respectively. Furthermore, the model was also able to predict therapeutic failure in 95.3% of patients using week 6 data and in 88% of cases using only pre-treatment data
Dulai et al., 2020 [54]	Response to therapy	A CDST was created for predicting treatment effectiveness of vedolizumab in CD using data from GEMINI 2 study	A linear relationship existed between CDST-defined groups, measured vedolizumab exposure, rapidity of onset of action and efficacy in GEMINI through week 52
Dulai et al., 2022 [55]	Response to therapy	A CDST identified patients with CD most likely to respond to vedolizumab and to predict real-world healthcare resource utilisation (HRU)	CDSTs identified lower rates of surgery or hospitalisation in CD patients with higher probability of vedolizumab response
Venkatapurapu et al., 2022 [56]	Response to therapy	A platform to predict endoscopic remission and mucosal healing after vedolizumab treatment	The model predicted endoscopic remission and mucosal healing for treatment with vedolizumab over 26 weeks, with overall sensitivities of 80% and 75% and overall specificities of 69% and 70%, respectively
Iacucci et al., 2023 [57]	Response to therapy	A model to predict response to biologics in IBD using pCLE in vivo and assess the binding of fluorescent-labelled biologics ex vivo	Higher mucosal binding of the drug target is associated with response to therapy in UC. In vivo, mucosal and microvascular changes detected by pCLE are associated with response to biologics in inflammatory bowel disease
	Advanced imaging		
Takenaka et al., 2021 [28]	Histological activity	In a previous study a deep neural network system based on endoscopic images of UC (DNUC) predicted histologic remission. In this follow-up study, it was evaluated if DNUC could predict patient prognosis	The DNUC could predict patient prognosis, and its predictive value was comparable with that of assessments by experts
	WLE		
Vande Casteele et al., 2022 [58]	Histological activity	A DL algorithm to quantify eosinophils in colonic biopsies	The model had sensitivity 0.86, specificity 0.91, accuracy 0.89
	WLE		
Reigle et al., 2024 [59]	Histological activity	Application of a DL to automate eosinophil counting	The inter-rater reliability was 0.96 (95% CI: 0.93–0.97). The correlation between two pathologists and the algorithm was 0.89 (95% CI: 0.82–0.94) and 0.88 (95% CI: 0.80–0.94), respectively
Ohara et al., 2022 [60]	Histological activity	DL-based models were trained to detect goblet cell mucus area from whole slide images of biopsy specimens	The model had sensitivity 0.83, specificity 0.99, accuracy 0.97
	WLE		
Iacucci et al., 2023 [61]	Histological activity	The PHRI was applied to a computer-assisted diagnostic system	When comparing the AI-generated assessment results with those generated by pathologists, the AI model was found to be highly sensitive and specific in determining the presence of neutrophils
	WLE-advanced imaging		
Gui et al., 2022 [62]	Histological activity	Evaluation of the applicability of the PHRI in an AI system on 614 biopsies from 307 UC patients	The algorithm showed a sensitivity of 78%, a specificity of 91.7%, and an accuracy of 86% in determining the presence or absence of neutrophils
	WLE-advanced imaging		
Zand et al., 2020 [63]	Response to therapy	Evaluation of a natural language processing (NLP)-based chatbot to categorise IBD patients’ electronic messages into various categories	The agreement between the algorithm and clinicians was 95%
Biasci et al., 2019 [64]	Risk stratification	ML models using RNA expression levels from whole blood samples to perform risk stratification	The ML model identified high- and low-risk groups for future dose escalations in both CD (75% vs. 35%) and UC (60% vs. 20%)
Cushing et al., 2019 [65]	Risk stratification	ML model created to predict 1-year relapse risk in CD after surgery, using non-invasive markers	Anti-TNF exposed patients with indolent postoperative courses were found to have a transcriptome signature distinct from those with aggressive disease
Stidham et al., 2021 [13]	Risk stratification	ML model developed to predict the risk of surgery in patients with CD	Anti-TNF therapy is the strongest predictor associated with a lower risk of surgery within 1 year
Dong et al., 2019 [66]	Risk stratification	ML model developed to predict the risk of surgery in patients with CD	Using variables such as age, sex, smoking status, perianal disease, previous surgical resection, the ML model showed higher accuracy and precision than the statistical model
Morilla et al., 2019 [67]	Response to therapy	A neural network was created that combining data from a pool of 3391 miRNA candidates and clinical factors in patients with ASUC	It effectively distinguished medical responders from non-responders with 97% accuracy; the miRNA-only model had 94% accuracy
Maeda et al., 2020 [68]	Detection of colonic neoplasm	Evaluation of an AI-assisted detection of colitis-associated neoplasms	The first case report in which an AI system detected colitis-associated neoplasms
	Advanced imaging		
Misawa et al., 2021 [69]	Detection of colonic neoplasm	Applications of EndoBrain-EYE in IBD patients	Two flat lesions with low-grade dysplasia were clearly highlighted by EndoBRAIN-EYE
	WLE		
Yamamoto et al., 2022 [70]	Detection of colonic neoplasm	Evaluation of an AI system for characterising neoplasia occurring in IBD	AI diagnostic accuracy of the AI model was higher than experts and non-experts (nonexperts, 77.8%; experts, 75.8%; AI model, 79.0%)
	WLE		
Rymarczyk et al., 2023 [71]	Histological activity	Evaluation of a DL models for automating histological assessments in IBD	AI-modelled GHAS and Geboes subgrades matched central readings with moderate to substantial agreement, with accuracies ranging from 65% to 89%
Matalka et al., 2013 [72]	Histological activity	Evaluation of a novel automated system to assess mucosal damage and architectural distortion in IBD	The developed system achieved an overall precision of 98.31%
Ohara et al., 2024 [73]	Histological activity	Evaluation of an AI system to detect neutrophils in UC biopsy specimens	The model achieved a performance of 0.77, 0.81, and 0.79 for precision, recall, and F-score, respectively
Del Amor et al., 2022 [74]	Histological activity	Evaluation of a novel MIL framework with location constraints able to determine the presence of UC activity based on neutrophils detection using WSI	In comparison with prior multiple instance learning settings, this method allowed for 10% improvements in accuracy
Peyrin Biroulet et al., 2024 [75]	Histological activity	Evaluation of an AI system to measure histological disease activity based on the Nancy index	The average ICC among the histopathologists was 89.3 and the average ICC between histopathologists and the AI tool was 87.2
Najdawi et al., 2023 [76]	Histological activity	Validation of CNN models that quantify histologic features in UC, directly from haematoxylin and eosin-stained whole slide images	The model accurately predicted Nancy histological index scores (*⍴* = 0.89, *p* < 0.001) when compared with pathologist consensus Nancy histological index scores. It also predicted histologic remission with a high accuracy of 0.97
Kiyokawa et al., 2022 [77]	Histological activity	Evaluation of a DL model to predict postoperative recurrence of CD by computational analysis of histopathologic images and to extract histologic characteristics associated with recurrence	The model achieved a highly accurate prediction of recurrence (area under the curve, 0.995
Rubin et al., 2024 [78]	Histological activity	Application of an AI tool based on DL to streamline the quantitative assessment of histopathology using the Nancy Index in UC	Confusion matrix analysis demonstrated an 80% correlation between predicted and true labels for Nancy scores of 0 or 4; a 96% correlation for a true score of 0 being predicted as 0 or 1; and a 100% correlation for a true score of 2 being predicted as 2 or 3
Vinsard et al., 2023 [79]	Detection of colonic neoplasm	Application of a CADe model of colorectal lesions in patients with IBD	IBD-CADe model on HDWLE had sensitivity, 95.1%; specificity, 98.8% and accuracy, 96.8%; and area under the curve, 0.85. IBD-CADe for chromoendoscopy images showed a sensitivity of 67.4%, specificity of 88.0%, accuracy of 77.8%, and area under the curve of 0.65
	WLE		
Abdelrahim et al., 2024 [80]	Detection of colonic neoplasm	AI model for lesion detection in IBD	The AI model had lesion detection rate, lesion per colonoscopy and neoplasia per colonoscopy of 90.4%, 4.6% and 0.96., respectively. The sensitivity and specificity of lesion characterisation were 87.5% and 80.6%, respectively
	WLE		
Stidham et al., 2021 [81]	Risk stratification	Evaluation of a ML models incorporating routinely collected laboratory studies to predict surgical outcomes in CD	The model achieved a mean area under the receiver operating characteristic of 0.78 (SD, 0.002). Anti-tumour necrosis factor use was the most influential predictor
Majumder et al., 2024 [82]	Risk stratification	This study aims to combine endocytoscope with intestinal barrier proteins assessment through ML-enabled multispectral spatial imaging to predict MAOs	The combination of endocytoscopy with Claudin-2 expression showed promise in accurately predicting MAOs over 12 months
	Advanced imaging		
Maeda et al., 2022 [83]	Risk stratification	Application of AI to predict clinical relapse of UC in clinical remission	The relapse rate was higher in the AI-Active group (28.4% [21/74]; 95% confidence interval, 18.5–40.1%) than in the AI-Healing group (4.9% [3/61]; 95% confidence interval, 1.0–13.7%; *p* < 0.001)
	Advanced imaging		
Omori et al., 2024 [84]	Risk stratification	Comparison between AI-assisted ultra-magnifying colonoscopy system for histological healing in UC and conventional light non-magnifying endoscopy	EndoBRAIN-UC showed a sensitivity of 74.2% and a specificity of 93.8% for histological diagnosis of remission
	WLE-advanced imaging		
Kuroki et al., 2024 [85]	Risk stratification	Evaluation of an AI-based system to diagnose “vascular-healing”	The clinical relapse rate was significantly higher in the AI-based vascular-active group (23.9% [16/67]) compared with the AI-based vascular-healing group (3.0% [1/33)]; *p* = 0.01)
	WLE		

ML machine learning; CD Crohn disease; UC ulcerative colitis; IBD inflammatory bowel disease; MES mayo endoscopic subscore; UCEIS ulcerative colitis endoscopic index of severity; RHI Robarts histopathology index; DL deep learning; CNN convoluted neural network; HD high definition; WLE white light endoscopy; VCE virtual chromoendoscopy; CE capsule endoscopy; ANN artificial neural network; CDST clinical decision support tool; CLE confocal laser endomicroscopy; PHRI simplified neutrophil-only Paddington International Chromoendoscopy Score virtual histological remission index; HR histological remission; SWE single-wavelength endoscopy; MIL multiple instance learning; WSI whole-slide images; ICC intraclass correlation coefficient; CADe computer-aided detection; MAO major adverse outcomes.

## References

[1] Abraham C., Cho J.H. (2009). Inflammatory bowel disease. N. Engl. J. Med..

[2] Kappelman M.D., Rifas-Shiman S.L., Kleinman K., Ollendorf D., Bousvaros A., Grand R.J., Finkelstein J.A. (2007). The prevalence and geographic distribution of Crohn’s disease and ulcerative colitis in the United States. Clin. Gastroenterol. Hepatol. Off. Clin. Pract. J. Am. Gastroenterol. Assoc..

[3] Loftus E.V. (2004). Clinical epidemiology of inflammatory bowel disease: Incidence, prevalence, and environmental influences. Gastroenterology.

[4] Herauf M., Coward S., Peña-Sánchez J.N., Bernstein C.N., Benchimol E.I., Kaplan G.G., Canadian Gastro-Intestinal Epidemiology Consortium (2024). Commentary on the Epidemiology of Inflammatory Bowel Disease in Compounding Prevalence Nations: Toward Sustaining Healthcare Delivery. Gastroenterology.

[5] Orlando A., Guglielmi F.W., Cottone M., Orlando E., Romano C., Sinagra E. (2013). Clinical implications of mucosal healing in the management of patients with inflammatory bowel disease. Dig. Liver Dis..

[6] Le Berre C., Danese S., Peyrin-Biroulet L. (2023). Can we change the natural course of inflammatory bowel disease?. Ther. Adv. Gastroenterol..

[7] Pakdin M., Zarei L., Bagheri Lankarani K., Ghahramani S. (2023). The cost of illness analysis of inflammatory bowel disease. BMC Gastroenterol..

[8] Maaser C., Sturm A., Vavricka S.R., Kucharzik T., Fiorino G., Annese V., Calabrese E., Baumgart D.C., Bettenworth D., Borralho Nunes P. (2019). ECCO-ESGAR Guideline for Diagnostic Assessment in IBD Part 1: Initial diagnosis, monitoring of known IBD, detection of complications. J. Crohns Colitis..

[9] Sturm A., Maaser C., Calabrese E., Annese V., Fiorino G., Kucharzik T., Vavricka S.R., Verstockt B., van Rheenen P., Tolan D. (2019). ECCO-ESGAR Guideline for Diagnostic Assessment in IBD Part 2: IBD scores and general principles and technical aspects. J. Crohns Colitis..

[10] Gu P., Mendonca O., Carter D., Dube S., Wang P., Huang X., Li D., Moore J.H., McGovern D.P.B. (2024). AI-luminating Artificial Intelligence in Inflammatory Bowel Diseases: A Narrative Review on the Role of AI in Endoscopy, Histology, and Imaging for IBD. Inflamm. Bowel Dis..

[11] Kaplan G.G., Windsor J.W. (2021). The four epidemiological stages in the global evolution of inflammatory bowel disease. Nat. Rev. Gastroenterol. Hepatol..

[12] Watermeyer G., Katsidzira L., Setshedi M., Devani S., Mudombi W., Kassianides C. (2022). Gastroenterology and Hepatology Association of sub-Saharan Africa (GHASSA). Inflammatory bowel disease in sub-Saharan Africa: Epidemiology, risk factors, and challenges in diagnosis. Lancet Gastroenterol. Hepatol..

[13] Stidham R.W., Takenaka K. (2022). Artificial Intelligence for Disease Assessment in Inflammatory Bowel Disease: How Will it Change Our Practice?. Gastroenterology.

[14] Da Rio L., Spadaccini M., Parigi T.L., Gabbiadini R., Dal Buono A., Busacca A., Maselli R., Fugazza A., Colombo M., Carrara S. (2023). Artificial intelligence and inflammatory bowel disease: Where are we going?. World J. Gastroenterol..

[15] Vamathevan J., Clark D., Czodrowski P., Dunham I., Ferran E., Lee G., Li B., Madabhushi A., Shah P., Spitzer M. (2019). Applications of machine learning in drug discovery and development. Nat. Rev. Drug. Discov..

[16] Ahmad H.A., East J.E., Panaccione R., Travis S., Canavan J.B., Usiskin K., Byrne M.F. (2023). Artificial Intelligence in Inflammatory Bowel Disease Endoscopy: Implications for Clinical Trials. J. Crohns Colitis..

[17] Ahmed M., Stone M.L., Stidham R.W. (2024). Artificial Intelligence and IBD: Where are We Now and Where Will We Be in the Future?. Curr. Gastroenterol. Rep..

[18] Yang Z.R. (2004). Biological applications of support vector machines. Brief. Bioinform..

[19] Rigatti S.J. (2017). Random Forest. J. Insur. Med..

[20] Sasaki Y., Hada R., Munakata A. (2003). Computer-aided grading system for endoscopic severity in patients with ulcerative colitis. Dig. Endosc..

[21] Kraszewski S., Szczurek W., Szymczak J., Reguła M., Neubauer K. (2021). Machine Learning Prediction Model for Inflammatory Bowel Disease Based on Laboratory Markers. Working Model in a Discovery Cohort Study. J. Clin. Med..

[22] Bossuyt P., Nakase H., Vermeire S., de Hertogh G., Eelbode T., Ferrante M., Hasegawa T., Willekens H., Ikemoto Y., Makino T. (2020). Automatic, computer-aided determination of endoscopic and histological inflammation in patients with mild to moderate ulcerative colitis based on red density. Gut.

[23] Stidham R.W., Liu W., Bishu S., Rice M.D., Higgins P.D.R., Zhu J., Nallamothu B.K., Waljee A.K. (2019). Performance of a Deep Learning Model vs Human Reviewers in Grading Endoscopic Disease Severity of Patients With Ulcerative Colitis. JAMA Netw. Open..

[24] Fan Y., Mu R., Xu H., Xie C., Zhang Y., Liu L., Wang L., Shi H., Hu Y., Ren J. (2023). Novel deep learning-based computer-aided diagnosis system for predicting inflammatory activity in ulcerative colitis. Gastrointest. Endosc..

[25] Ozawa T., Ishihara S., Fujishiro M., Saito H., Kumagai Y., Shichijo S., Aoyama K., Tada T. (2019). Novel computer-assisted diagnosis system for endoscopic disease activity in patients with ulcerative colitis. Gastrointest. Endosc..

[26] Takabayashi K., Kobayashi T., Matsuoka K., Levesque B.G., Kawamura T., Tanaka K., Kadota T., Bise R., Uchida S., Kanai T. (2024). Artificial intelligence quantifying endoscopic severity of ulcerative colitis in gradation scale. Dig. Endosc. Off. J. Jpn. Gastroenterol. Endosc. Soc..

[27] Takenaka K., Ohtsuka K., Fujii T., Negi M., Suzuki K., Shimizu H., Oshima S., Akiyama S., Motobayashi M., Nagahori M. (2020). Development and Validation of a Deep Neural Network for Accurate Evaluation of Endoscopic Images From Patients With Ulcerative Colitis. Gastroenterology.

[28] Takenaka K., Ohtsuka K., Fujii T., Oshima S., Okamoto R., Watanabe M. (2021). Deep Neural Network Accurately Predicts Prognosis of Ulcerative Colitis Using Endoscopic Images. Gastroenterology.

[29] Yao H., Najarian K., Gryak J., Bishu S., Rice M.D., Waljee A.K., Wilkins H.J., Stidham R.W. (2021). Fully automated endoscopic disease activity assessment in ulcerative colitis. Gastrointest. Endosc..

[30] Gottlieb K., Requa J., Karnes W., Chandra Gudivada R., Shen J., Rael E., Arora V., Dao T., Ninh A., McGill J. (2021). Central Reading of Ulcerative Colitis Clinical Trial Videos Using Neural Networks. Gastroenterology.

[31] Iacucci M., Cannatelli R., Parigi T.L., Nardone O.M., Tontini G.E., Labarile N., Buda A., Rimondi A., Bazarova A., Bisschops R. (2023). A virtual chromoendoscopy artificial intelligence system to detect endoscopic and histologic activity/remission and predict clinical outcomes in ulcerative colitis. Endoscopy.

[32] Charisis V.S., Hadjileontiadis L.J. (2016). Potential of hybrid adaptive filtering in inflammatory lesion detection from capsule endoscopy images. World J. Gastroenterol..

[33] Fan S., Xu L., Fan Y., Wei K., Li L. (2018). Computer-aided detection of small intestinal ulcer and erosion in wireless capsule endoscopy images. Phys. Med. Biol..

[34] Afonso J., Saraiva M.M., Ferreira J.P.S., Cardoso H., Ribeiro T., Andrade P., Parente M., Jorge R.N., Macedo G. (2022). Automated detection of ulcers and erosions in capsule endoscopy images using a convolutional neuralnetwork. Med. Biol. Eng. Comput..

[35] Aoki T., Yamada A., Aoyama K., Saito H., Tsuboi A., Nakada A., Niikura R., Fujishiro M., Oka S., Ishihara S. (2019). Automatic detection of erosions and ulcerations in wireless capsule endoscopy images based on a deep convolutional neural network. Gastrointest. Endosc..

[36] Ferreira J.P.S., de Mascarenhas Saraiva M.J.Q.E.C., Afonso J.P.L., Ribeiro T.F.C., Cardoso H.M.C., Ribeiro Andrade A.P., de Mascarenhas Saraiva M.N.G., Parente M.P.L., Natal Jorge R., Lopes S.I.O. (2022). Identification of Ulcers and Erosions by the Novel PillcamTM Crohn’s Capsule Using a Convolutional Neural Network: A Multicentre Pilot Study. J. Crohns Colitis..

[37] Kratter T., Shapira N., Lev Y., Mauda O., Moshkovitz Y., Shitrit R., Konyo S., Ukashi O., Dar L., Shlomi O. (2022). Deep Learning Multi-Domain Model Provides Accurate Detection and Grading of Mucosal Ulcers in Different Capsule Endoscopy Types. Diagnostic.

[38] Brodersen J.B., Jensen M.D., Leenhardt R., Kjeldsen J., Histace A., Knudsen T., Dray X. (2024). Artificial Intelligence-assisted Analysis of Pan-enteric Capsule Endoscopy in Patients with Suspected Crohn’s Disease: A Study on Diagnostic Performance. J. Crohns Colitis..

[39] Klang E., Grinman A., Soffer S., Margalit Yehuda R., Barzilay O., Amitai M.M., Konen E., Ben-Horin S., Eliakim R., Barash Y. (2021). Automated Detection of Crohn’s Disease Intestinal Strictures on Capsule Endoscopy Images Using Deep Neural Networks. J. Crohns Colitis..

[40] Barash Y., Azaria L., Soffer S., Margalit Yehuda R., Shlomi O., Ben-Horin S., Eliakim R., Klang E., Kopylov U. (2021). Ulcer severity grading in video capsule images of patients with Crohn’s disease: An ordinal neural network solution. Gastrointest. Endosc..

[41] Ding Z., Shi H., Zhang H., Meng L., Fan M., Han C., Zhang K., Ming F., Xie X., Liu H. (2019). Gastroenterologist-Level Identification of Small-Bowel Diseases and Normal Variants by Capsule Endoscopy Using a Deep-Learning Model. Gastroenterology.

[42] Aoki T., Yamada A., Aoyama K., Saito H., Fujisawa G., Odawara N., Kondo R., Tsuboi A., Ishibashi R., Nakada A. (2020). Clinical usefulness of a deep learning-based system as the first screening on small-bowel capsule endoscopy reading. Dig. Endosc. Off. J. Jpn. Gastroenterol. Endosc. Soc..

[43] Quénéhervé L., David G., Bourreille A., Hardouin J.B., Rahmi G., Neunlist M., Brégeon J., Coron E. (2019). Quantitative assessment of mucosal architecture using computer-based analysis of confocal laser endomicroscopy in inflammatory bowel diseases. Gastrointest. Endosc..

[44] Maeda Y., Kudo S.E., Mori Y., Misawa M., Ogata N., Sasanuma S., Wakamura K., Oda M., Mori K., Ohtsuka K. (2019). Fully automated diagnostic system with artificial intelligence using endocytoscopy to identify the presence of histologic inflammation associated with ulcerative colitis (with video). Gastrointest. Endosc..

[45] Bossuyt P., De Hertogh G., Eelbode T., Vermeire S., Bisschops R. (2021). Computer-Aided Diagnosis With Monochromatic Light Endoscopy for Scoring Histologic Remission in Ulcerative Colitis. Gastroenterolog.

[46] Sinonquel P., Lenfant M., Eelbode T., Watanabe H., Callaerts B., Bossuyt P., Verstockt B., Sabino J.P.G., De Hertogh G., Maes F. (2024). Development of an Automated Tool for the Estimation of Histological Remission in Ulcerative Colitis Using Single-Wavelength Endoscopy Technology. J. Crohns Colitis..

[47] Con D., van Langenberg D.R., Vasudevan A. (2021). Deep learning vs conventional learning algorithms for clinical prediction in Crohn’s disease: A proof-of-concept study. World J. Gastroenterol..

[48] Popa I.V., Burlacu A., Mihai C., Prelipcean C.C. (2020). A Machine Learning Model Accurately Predicts Ulcerative Colitis Activity at One Year in Patients Treated with Anti-Tumour Necrosis Factor α Agents. Med. Kaunas. Lith..

[49] Park S.K., Kim Y.B., Kim S., Lee C.W., Choi C.H., Kang S.B., Kim T.O., Bang K.B., Chun J., Cha J.M. (2022). Development of a Machine Learning Model to Predict Non-Durable Response to Anti-TNF Therapy in Crohn’s Disease Using Transcriptome Imputed from Genotypes. J. Pers. Med..

[50] Waljee A.K., Wallace B.I., Cohen-Mekelburg S., Liu Y., Liu B., Sauder K., Stidham R.W., Zhu J., Higgins P.D.R. (2019). Development and Validation of Machine Learning Models in Prediction of Remission in Patients With Moderate to Severe Crohn Disease. JAMA Netw. Open..

[51] He M., Li C., Tang W., Kang Y., Zuo Y., Wang Y. (2021). Machine learning gene expression predicting model for ustekinumab response in patients with Crohn’s disease. Immun. Inflamm. Dis..

[52] Waljee A.K., Sauder K., Patel A., Segar S., Liu B., Zhang Y., Zhu J., Stidham R.W., Balis U., Higgins P.D.R. (2017). Machine Learning Algorithms for Objective Remission and Clinical Outcomes with Thiopurines. J. Crohns Colitis..

[53] Waljee A.K., Liu B., Sauder K., Zhu J., Govani S.M., Stidham R.W., Higgins P.D.R. (2018). Predicting Corticosteroid-Free Biologic Remission with Vedolizumab in Crohn’s Disease. Inflamm. Bowel Dis..

[54] Dulai P.S., Amiot A., Peyrin-Biroulet L., Jairath V., Serrero M., Filippi J., Singh S., Pariente B., Loftus E.V., Roblin X. (2020). A clinical decision support tool may help to optimise vedolizumab therapy in Crohn’s disease. Aliment. Pharmacol. Ther..

[55] Dulai P.S., Wan Y., Huang Z., Luo M. (2022). Probability of Response as Defined by a Clinical Decision Support Tool Is Associated With Lower Healthcare Resource Utilization in Vedolizumab-Treated Patients With Crohn’s Disease. Crohns. Colitis 360.

[56] Venkatapurapu S.P., Iwakiri R., Udagawa E., Patidar N., Qi Z., Takayama R., Kumar K., Sato Y., Behar M., Offner P. (2022). A Computational Platform Integrating a Mechanistic Model of Crohn’s Disease for Predicting Temporal Progression of Mucosal Damage and Healing. Adv. Ther..

[57] Iacucci M., Jeffery L., Acharjee A., Grisan E., Buda A., Nardone O.M., Smith S.C.L., Labarile N., Zardo D., Ungar B. (2023). Computer-Aided Imaging Analysis of Probe-Based Confocal Laser Endomicroscopy With Molecular Labeling and Gene Expression Identifies Markers of Response to Biological Therapy in IBD Patients: The Endo-Omics Study. Inflamm. Bowel Dis..

[58] Vande Casteele N., Leighton J.A., Pasha S.F., Cusimano F., Mookhoek A., Hagen C.E., Rosty C., Pai R.K., Pai R.K. (2022). Utilizing Deep Learning to Analyze Whole Slide Images of Colonic Biopsies for Associations Between Eosinophil Density and Clinicopathologic Features in Active Ulcerative Colitis. Inflamm. Bowel Dis..

[59] Reigle J., Lopez-Nunez O., Drysdale E., Abuquteish D., Liu X., Putra J., Erdman L., Griffiths A.M., Prasath S., Siddiqui I. (2024). Using Deep Learning to Automate Eosinophil Counting in Pediatric Ulcerative Colitis Histopathological Images. MedRxiv..

[60] Ohara J., Nemoto T., Maeda Y., Ogata N., Kudo S.E., Yamochi T. (2022). Deep learning-based automated quantification of goblet cell mucus using histological images as a predictor of clinical relapse of ulcerative colitis with endoscopic remission. J. Gastroenterol..

[61] Iacucci M., Parigi T.L., Del Amor R., Meseguer P., Mandelli G., Bozzola A., Bazarova A., Bhandari P., Bisschops R., Danese S. (2023). Artificial Intelligence Enabled Histological Prediction of Remission or Activity and Clinical Outcomes in Ulcerative Colitis. Gastroenterology.

[62] Gui X., Bazarova A., Del Amor R., Vieth M., de Hertogh G., Villanacci V., Zardo D., Parigi T.L., Røyset E.S., Shivaji U.N. (2022). PICaSSO Histologic Remission Index (PHRI) in ulcerative colitis: Development of a novel simplified histological score for monitoring mucosal healing and predicting clinical outcomes and its applicability in an artificial intelligence system. Gut.

[63] Zand A., Sharma A., Stokes Z., Reynolds C., Montilla A., Sauk J., Hommes D. (2020). An Exploration Into the Use of a Chatbot for Patients With Inflammatory Bowel Diseases: Retrospective Cohort Study. J. Med. Internet. Res..

[64] Biasci D., Lee J.C., Noor N.M., Pombal D.R., Hou M., Lewis N., Ahmad T., Hart A., Parkes M., McKinney E.F. (2019). A blood-based prognostic biomarker in IBD. Gut.

[65] Cushing K.C., Mclean R., McDonald K.G., Gustafsson J.K., Knoop K.A., Kulkarni D.H., Sartor R.B., Newberry R.D. (2019). Predicting Risk of Postoperative Disease Recurrence in Crohn’s Disease: Patients With Indolent Crohn’s Disease Have Distinct Whole Transcriptome Profiles at the Time of First Surgery. Inflamm. Bowel Dis..

[66] Dong Y., Xu L., Fan Y., Xiang P., Gao X., Chen Y., Zhang W., Ge Q. (2019). A novel surgical predictive model for Chinese Crohn’s disease patients. Medicine.

[67] Morilla I., Uzzan M., Laharie D., Cazals-Hatem D., Denost Q., Daniel F., Belleannee G., Bouhnik Y., Wainrib G., Panis Y. (2019). Colonic MicroRNA Profiles, Identified by a Deep Learning Algorithm, That Predict Responses to Therapy of Patients With Acute Severe Ulcerative Colitis. Clin. Gastroenterol. Hepatol. Off. Clin. Pract. J. Am. Gastroenterol. Assoc..

[68] Maeda Y., Kudo S.E., Ogata N., Misawa M., Mori Y., Mori K., Ohtsuka K. (2021). Can artificial intelligence help to detect dysplasia in patients with ulcerative colitis?. Endoscopy.

[69] Misawa M., Kudo S.E., Mori Y., Hotta K., Ohtsuka K., Matsuda T., Saito S., Kudo T., Baba T., Ishida F. (2021). Development of a computer-aided detection system for colonoscopy and a publicly accessible large colonoscopy video database (with video). Gastrointest. Endosc..

[70] Yamamoto S., Kinugasa H., Hamada K., Tomiya M., Tanimoto T., Ohto A., Toda A., Takei D., Matsubara M., Suzuki S. (2022). The diagnostic ability to classify neoplasias occurring in inflammatory bowel disease by artificial intelligence and endoscopists: A pilot study. J. Gastroenterol. Hepatol..

[71] Rymarczyk D., Schultz W., Borowa A., Friedman J.R., Danel T., Branigan P., Chałupczak M., Bracha A., Krawiec T., Warchoł M. (2024). Deep Learning Models Capture Histological Disease Activity in Crohn’s Disease and Ulcerative Colitis with High Fidelity. J. Crohns Colitis..

[72] Matalka I.I., Al-Omari F.A., Salama R.M., Mohtaseb A.H. (2013). A novel approach for quantitative assessment of mucosal damage in inflammatory bowel disease. Diagn. Pathol..

[73] Ohara J., Maeda Y., Ogata N., Kuroki T., Misawa M., Kudo S.E., Nemoto T., Yamochi T., Iacucci M. (2024). Automated Neutrophil Quantification and Histological Score Estimation in Ulcerative Colitis. Clin. Gastroenterol. Hepatol. Off. Clin. Pract. J. Am. Gastroenterol. Assoc..

[74] Del Amor R., Meseguer P., Parigi T.L., Villanacci V., Colomer A., Launet L., Bazarova A., Tontini G.E., Bisschops R., de Hertogh G. (2022). Constrained multiple instance learning for ulcerative colitis prediction using histological images. Comput. Methods Programs Biomed..

[75] Peyrin-Biroulet L., Adsul S., Stancati A., Dehmeshki J., Kubassova O. (2024). An artificial intelligence-driven scoring system to measure histological disease activity in ulcerative colitis. United Eur. Gastroenterol. J..

[76] Najdawi F., Sucipto K., Mistry P., Hennek S., Jayson C.K.B., Lin M., Fahy D., Kinsey S., Wapinski I., Beck A.H. (2023). Artificial Intelligence Enables Quantitative Assessment of Ulcerative Colitis Histology. Mod. Pathol. Off..

[77] Kiyokawa H., Abe M., Matsui T., Kurashige M., Ohshima K., Tahara S., Nojima S., Ogino T., Sekido Y., Mizushima T. (2022). Deep Learning Analysis of Histologic Images from Intestinal Specimen Reveals Adipocyte Shrinkage and Mast Cell Infiltration to Predict Postoperative Crohn Disease. Am. J. Pathol..

[78] Rubin D.T., Kubassova O., Weber C.R., Adsul S., Freire M., Biedermann L., Koelzer V.H., Bressler B., Xiong W., Niess J.H. (2024). Deployment of an Artificial Intelligence Histology Tool to Aid Qualitative Assessment of Histopathology Using the Nancy Histopathology Index in Ulcerative Colitis. Inflamm. Bowel Dis..

[79] Guerrero Vinsard D., Fetzer J.R., Agrawal U., Singh J., Damani D.N., Sivasubramaniam P., Poigai Arunachalam S., Leggett C., Raffals L.E., Coelho-Prabhu N. (2023). Development of an artificial intelligence tool for detecting colorectal lesions in inflammatory bowel disease. iGIE.

[80] Abdelrahim M., Siggens K., Iwadate Y., Maeda N., Htet H., Bhandari P. (2024). New AI model for neoplasia detection and characterisation in inflammatory bowel disease. Gut.

[81] Stidham R.W., Liu Y., Enchakalody B., Van T., Krishnamurthy V., Su G.L., Zhu J., Waljee A.K. (2021). The Use of Readily Available Longitudinal Data to Predict the Likelihood of Surgery in Crohn Disease. Inflamm. Bowel Dis..

[82] Majumder S., Santacroce G., Maeda Y., Zammarchi I., Puga-Tejada M., Ditonno I., Hayes B., Crotty R., Fennell E., Shivaji U.N. (2024). Endocytoscopy with automated multispectral intestinal barrier pathology imaging for assessment of deep healing to predict outcomes in ulcerative colitis. Gut.

[83] Maeda Y., Kudo S.E., Ogata N., Misawa M., Iacucci M., Homma M., Nemoto T., Takishima K., Mochida K., Miyachi H. (2022). Evaluation in real-time use of artificial intelligence during colonoscopy to predict relapse of ulcerative colitis: A prospective study. Gastrointest. Endosc..

[84] Omori T., Yamamoto T., Murasugi S., Koroku M., Yonezawa M., Nonaka K., Nagashima Y., Nakamura S., Tokushige K. (2024). Comparison of Endoscopic and Artificial Intelligence Diagnoses for Predicting the Histological Healing of Ulcerative Colitis in a Real-World Clinical Setting. Crohns Colitis..

[85] Kuroki T., Maeda Y., Kudo S.E., Ogata N., Iacucci M., Takishima K., Ide Y., Shibuya T., Semba S., Kawashima J. (2024). A novel artificial intelligence-assisted “vascular healing” diagnosis for prediction of future clinical relapse in patients with ulcerative colitis: A prospective cohort study (with video). Gastrointest. Endosc..

[86] Turner D., Ricciuto A., Lewis A., D’Amico F., Dhaliwal J., Griffiths A.M., Bettenworth D., Sandborn W.J., Sands B.E., Reinisch W. (2021). International Organization for the Study of IBD. STRIDE-II: An Update on the Selecting Therapeutic Targets in Inflammatory Bowel Disease (STRIDE) Initiative of the International Organization for the Study of IBD (IOIBD): Determining Therapeutic Goals for Treat-to-Target strategies in IBD. Gastroenterology.

[87] Hashash J.G., Yu Ci Ng F., Farraye F.A., Wang Y., Colucci D.R., Baxi S., Muneer S., Reddan M., Shingru P., Melmed G.Y. (2024). Inter- and Intraobserver Variability on Endoscopic Scoring Systems in Crohn’s Disease and Ulcerative Colitis: A Systematic Review and Meta-Analysis. Inflamm. Bowel Dis..

[88] Stidham R.W., Cai L., Cheng S., Rajaei F., Hiatt T., Wittrup E., Rice M.D., Bishu S., Wehkamp J., Schultz W. (2024). Using Computer Vision to Improve Endoscopic Disease Quantification in Therapeutic Clinical Trials of Ulcerative Colitis. Gastroenterology.

[89] Takenaka K., Fujii T., Kawamoto A., Suzuki K., Shimizu H., Maeyashiki C., Yamaji O., Motobayashi M., Igarashi A., Hanazawa R. (2022). Deep neural network for video colonoscopy of ulcerative colitis: A cross-sectional study. Lancet Gastroenterol. Hepatol..

[90] Rimondi A., Gottlieb K., Despott E.J., Iacucci M., Murino A., Tontini G.E. (2024). Can artificial intelligence replace endoscopists when assessing mucosal healing in ulcerative colitis? A systematic review and diagnostic test accuracy meta-analysis. Dig. Liver Dis. Off. J. Ital. Soc. Gastroenterol. Ital. Assoc. Study Liver.

[91] Lv B., Ma L., Shi Y., Tao T., Shi Y. (2023). A systematic review and meta-analysis of artificial intelligence-diagnosed endoscopic remission in ulcerative colitis. iScience.

[92] Jahagirdar V., Bapaye J., Chandan S., Ponnada S., Kochhar G.S., Navaneethan U., Mohan B.P. (2023). Diagnostic accuracy of convolutional neural network-based machine learning algorithms in endoscopic severity prediction of ulcerative colitis: A systematic review and meta-analysis. Gastrointest. Endosc..

[93] Sipponen T., Nuutinen H., Turunen U., Färkkilä M. (2010). Endoscopic evaluation of Crohn’s disease activity: Comparison of the CDEIS and the SES-CD. Inflamm. Bowel Dis..

[94] Leenhardt R., Buisson A., Bourreille A., Marteau P., Koulaouzidis A., Li C., Keuchel M., Rondonotti E., Toth E., Plevris J.N. (2020). Nomenclature and semantic descriptions of ulcerative and inflammatory lesions seen in Crohn’s disease in small bowel capsule endoscopy: An international Delphi consensus statement. United Eur. Gastroenterol. J..

[95] Mohan B.P., Khan S.R., Kassab L.L., Ponnada S., Chandan S., Ali T., Dulai P.S., Adler D.G., Kochhar G.S. (2021). High pooled performance of convolutional neural networks in computer-aided diagnosis of GI ulcers and/or hemorrhage on wireless capsule endoscopy images: A systematic review and meta-analysis. Gastrointest. Endosc..

[96] Nguyen N.H., Picetti D., Dulai P.S., Jairath V., Sandborn W.J., Ohno-Machado L., Chen P.L., Singh S. (2022). Machine Learning-based Prediction Models for Diagnosis and Prognosis in Inflammatory Bowel Diseases: A Systematic Review. J. Crohns Colitis..

[97] Gordon H., Minozzi S., Kopylov U., Verstockt B., Chaparro M., Buskens C., Warusavitarne J., Agrawal M., Allocca M., Atreya R. (2024). ECCO Guidelines on Therapeutics in Crohn’s Disease: Medical Treatment. J. Crohns Colitis..

[98] Brooks-Warburton J., Ashton J., Dhar A., Tham T., Allen P.B., Hoque S., Lovat L.B., Sebastian S. (2022). Artificial intelligence and inflammatory bowel disease: Practicalities and future prospects. Frontline Gastroenterol..

[99] Yin A.L., Hachuel D., Pollak J.P., Scherl E.J., Estrin D. (2019). Digital Health Apps in the Clinical Care of Inflammatory Bowel Disease: Scoping Review. J. Med. Internet. Res..

[100] Jagannath B., Muthukumar S., Prasad S. (2021). Wearable Sweat Sensing Device For Detection Of Ibd Biomarkers. Inflamm. Bowel Dis..

[101] Cross R.K., Langenberg P., Regueiro M., Schwartz D.A., Tracy J.K., Collins J.F., Katz J., Ghazi L., Patil S.A., Quezada S.M. (2019). A Randomized Controlled Trial of TELEmedicine for Patients with Inflammatory Bowel Disease (TELE-IBD). Am. J. Gastroenterol..

[102] Puga-Tejada M., Majumder S., Maeda Y., Zammarchi I., Ditonno I., Santacroce G., Capobianco I., Robles-Medranda C., Ghosh S., Iacucci M. (2025). Artificial intelligence–enabled histology exhibits comparable accuracy to pathologists in assessing histological remission in ulcerative colitis: A systematic review, meta-analysis, and meta-regression. J. Crohns Colitis..

[103] Bajwa J., Munir U., Nori A., Williams B. (2021). Artificial intelligence in healthcare: Transforming the practice of medicine. Futur. Healthc. J..

[104] Liu X., Faes L., Kale A.U., Wagner S.K., Fu D.J., Bruynseels A., Mahendiran T., Moraes G., Shamdas M., Kern C. (2019). A comparison of deep learning performance against health-care professionals in detecting diseases from medical imaging: A systematic review and meta-analysis. Lancet Digit. Health..

[105] Kröner P.T., Engels M.M., Glicksberg B.S., Johnson K.W., Mzaik O., van Hooft J.E., Wallace M.B., El-Serag H.B., Krittanawong C. (2021). Artificial intelligence in gastroenterology: A state-of-the-art review. World J. Gastroenterol..

[106] Linardatos P., Papastefanopoulos V., Kotsiantis S. (2020). Explainable AI: A Review of Machine Learning Interpretability Methods. Entropy.

[107] Tontini G.E., Rimondi A., Vernero M., Neumann H., Vecchi M., Bezzio C., Cavallaro F. (2021). Artificial intelligence in gastrointestinal endoscopy for inflammatory bowel disease: A systematic review and new horizons. Ther. Adv. Gastroenterol..

[108] Sedano R., Hogan M., McDonald C., Aswani-Omprakash T., Ma C., Jairath V. (2022). Underrepresentation of Minorities and Lack of Race Reporting in Ulcerative Colitis Drug Development Clinical Trials. Inflamm. Bowel Dis..

[109] Sedano R., Hogan M., Mcdonald C., Aswani-Omprakash T., Ma C., Jairath V. (2022). Underrepresentation of Minorities and Underreporting of Race and Ethnicity in Crohn’s Disease Clinical Trials. Gastroenterology.

[110] Shah S., Shillington A.C., Kabagambe E.K., Deering K.L., Babin S., Capelouto J., Pulliam C., Patel A., LaChappelle B., Liu J. (2024). Racial and Ethnic Disparities in Patients With Inflammatory Bowel Disease: An Online Survey. Inflamm. Bowel Dis..

[111] Liu X., Cruz Rivera S., Moher D., Calvert M.J., Denniston A.K., SPIRIT-AI and CONSORT-AI Working Group (2020). Reporting guidelines for clinical trial reports for interventions involving artificial intelligence: The CONSORT-AI extension. Lancet Digit. Health..

[112] Ehsani-Moghaddam B., Martin K., Queenan J.A. (2021). Data quality in healthcare: A report of practical experience with the Canadian Primary Care Sentinel Surveillance Network data. Health Inf. Manag. J. Health Inf. Manag. Assoc. Aust..

[113] Nazer L.H., Zatarah R., Waldrip S., Chen Ke J.X., Moukheiber M., Khanna A.K., Hicklen R.S., Moukheiber L., Moukheiber D., Ma H. (2023). Bias in artificial intelligence algorithms and recommendations for mitigation. PLoS Digit. Health.

[114] Yang J., Soltan A.A.S., Eyre D.W., Clifton D.A. (2023). Algorithmic fairness and bias mitigation for clinical machine learning with deep reinforcement learning. Nat. Mach. Intell..

[115] Health C for D and R. (2025). Artificial Intelligence and Machine Learning in Software as a Medical Device.

[116] Davenport T., Kalakota R. (2019). The potential for artificial intelligence in healthcare. Future Healthc. J..

[117] Fraser A.G., Biasin E., Bijnens B., Bruining N., Caiani E.G., Cobbaert K., Davies R.H., Gilbert S.H., Hovestandt L., Kamenjasevic E. (2023). Artificial intelligence in medical device software and high-risk medical devices-a review of definitions, expert recommendations and regulatory initiatives. Expert. Rev. Med. Devices..

[118] Sedano R., Solitano V., Vuyyuru S.K., Yuan Y., Hanžel J., Ma C., Nardone O.M., Jairath V. (2025). Artificial intelligence to revolutionize IBD clinical trials: A comprehensive review. Ther. Adv. Gastroenterol..

